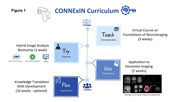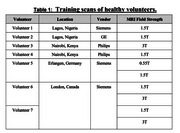# Comprehensive Neuroimaging Analysis Experience In Resource Constrained settings (CONNExIN): An Approach to Advance Dementia Neuroimaging Training in LMICs

**DOI:** 10.1002/alz70862_109800

**Published:** 2025-12-23

**Authors:** Ethan Draper, Jasmine Cakmak, Kesavi Kanagasabai, Harrison Aduluwa, Alfonso Fajardo, Oluwateniola Akinwale, Cristian Montalba, Jonathan Gallego Rudolf, Channelle Tham, Guy Poloni, Jackline Thairu, Njideka U Okubadejo, Fatade Abiodun, Sheila Waa, Thomas Thesen, Chinedu T Udeh‐Momoh, Farouk Dako, Udunna Anazodo

**Affiliations:** ^1^ Montreal Neurological Institute, Montreal, QC Canada; ^2^ Montreal Neurological Institute, McGill University, Montreal, QC Canada; ^3^ Department of Medical Biophysics, Western University, London, ON Canada; ^4^ Douglas Mental Health University Institute, Centre for Studies on the Prevention of Alzheimer's Disease (StoP‐AD), Montréal, QC Canada; ^5^ Johns Hopkins University, Baltimore, MD USA; ^6^ Biomedical Imaging Center, Pontificia Universidad Católica de Chile, Santiago, Santiago Chile; ^7^ Douglas Research Centre, McGill University, Montreal, QC Canada; ^8^ Radboud University, Nijmegen Netherlands; ^9^ Siemens Healtineers, Erlangen Germany; ^10^ Sonar Imaging Centre, Nairobi Kenya; ^11^ College of Medicine, University of Lagos, Lagos Nigeria; ^12^ Crestview Radiology Ltd, Lagos Nigeria; ^13^ Aga Khan University, Nairobi Kenya; ^14^ New York University, School of Medicine, New York, NY USA; ^15^ Brain and Mind Institute, Aga Khan University, Nairobi Kenya; ^16^ Wake Forest University School of Medicine, Winston‐Salem, NC USA; ^17^ Aga Khan University Brain and Mind Institute, Nairobi, Nairobi Kenya; ^18^ Perelman School of Medicine, University of Pennsylvania, Philadelphia, PA USA; ^19^ Medical Artificial Intelligence (MAI) Laboratory, Crestview Radiology Limited, Lagos Nigeria

## Abstract

**Background:**

Positron emission tomography (PET) and magnetic resonance imaging (MRI) are established imaging technologies for dementia management in both clinical (e.g., diagnosis) and research (e.g., identifying biomarkers) settings. Though still far below global rates, access to PET and MRI in low‐ and middle‐income countries (LMICs) is improving. However, there remains a dearth of research personnel in LMICs trained to use neuroimaging data for research.

**Method:**

CONNExIN (*COmprehensive Neuroimaging aNalysis Experience In resource constraiNed settings*) is a hybrid initiative led by Montreal Neurological Institute, McGill University, in collaboration with AFRICA‐FINGERS [1], and the Consortium for Advancement of MRI Education and Research in Africa (CAMERA) [2]. CONNExIN [3] implements RAD‐AID’s Teach‐Try‐Use strategy, previously applied by CAMERA to improve MRI and PET analysis competencies through seminars and hands‐on skills development [4] (Figure 1). Multi‐scanner (0.55T, 1.5T, and 3T) brain MRI data were acquired on seven healthy volunteers at six sites including at AFRICA‐FINGERS sites (Lagos and Nairobi) and used strictly for training (Table 1).

**Result:**

This 16‐week program began on August 26th, 2024 (Figure 1). Following three weeks of self‐paced virtual content and weekend tutorials, 34 African students and clinicians from 6 countries completed a hybrid bootcamp. The one‐week bootcamp included on‐site training hosted at Crestview Radiology (Lagos, Nigeria) and Aga Khan University (Nairobi, Kenya) and virtual participation. Participants analyzed local (Table 1) and open‐access brain scans (PREVENT‐AD) [5] in groups, self‐selecting one of six modalities (i.e., MRI and PET). At the end of the program in December 2024, a total of 120 hours of no‐cost neuroimaging analysis and dementia research training was provided including design, implementation, and dissemination. Participants are currently being guided on science communication through drafting and submitting conference abstracts including to AAIC. All training materials will be shared on protocols.io for wider dissemination.

**Conclusion:**

CONNExIN is training a cohort to analyze neuroimaging data and become local experts who can train others, thereby improving dementia imaging research capacity in LMICs.

[1] Udeh‐Momoh CT, et al. Alzheimers Dement. 2024

[2] https://www.cameramriafrica.org/

[3] event.fourwaves.com/connexin

[4] Mumuni AN, et al. J Am Coll Radiol. 2024

[5] Tremblay‐Mercier J, et al. Neuroimage Clin. 2021